# Spatial and Temporal Clustering of Chikungunya Virus Transmission in Dominica

**DOI:** 10.1371/journal.pntd.0003977

**Published:** 2015-08-14

**Authors:** Elaine O. Nsoesie, R. Paul Ricketts, Heidi E. Brown, Durland Fish, David P. Durham, Martial L. Ndeffo Mbah, Trudy Christian, Shalauddin Ahmed, Clement Marcellin, Ellen Shelly, Katharine Owers, Natasha Wenzel, Alison P. Galvani, John S. Brownstein

**Affiliations:** 1 Children’s Hospital Informatics Program, Boston Children’s Hospital, Boston, Massachusetts, United States of America; 2 Department of Pediatrics, Harvard Medical School, Boston, Massachusetts, United States of America; 3 Institute for Health Metrics and Evaluation, University of Washington, Seattle, Washington, United States of America; 4 Health Information Unit, Ministry of Health, Roseau, Commonwealth of Dominica; 5 Division of Epidemiology and Biostatistics, Mel and Enid Zuckerman College of Public Health, University of Arizona, Tucson, Arizona, United States of America; 6 Department of Epidemiology of Microbial Disease, Yale School of Public Health, New Haven, Connecticut, United States of America; 7 Center for Infectious Disease Modeling and Analysis, Yale School of Public Health, New Haven, Connecticut, United States of America; 8 Environmental Health Department, Ministry of Health, Roseau, Commonwealth of Dominica; 9 Department of Epidemiology, Biostatistics and Occupational Health, McGill University, Montreal, Quebec, Canada; Oswaldo Cruz Foundation, BRAZIL

## Abstract

Using geo-referenced case data, we present spatial and spatio-temporal cluster analyses of the early spread of the 2013–2015 chikungunya virus (CHIKV) in Dominica, an island in the Caribbean. Spatial coordinates of the locations of the first 417 reported cases observed between December 15^th^, 2013 and March 11^th^, 2014, were captured using the Global Positioning System (GPS). We observed a preponderance of female cases, which has been reported for CHIKV outbreaks in other regions. We also noted statistically significant spatial and spatio-temporal clusters in highly populated areas and observed major clusters prior to implementation of intensive vector control programs suggesting early vector control measures, and education had an impact on the spread of the CHIKV epidemic in Dominica. A dynamical identification of clusters can lead to local assessment of risk and provide opportunities for targeted control efforts for nations experiencing CHIKV outbreaks.

## Author Summary

Chikungunya is a disease transmitted by mosquitoes. Currently, there is an epidemic of chikungunya in several islands and countries in the Americas. Despite efforts at understanding and predicting spread, there have been no studies assessing the spatio-temporal spread of chikungunya in any of the Caribbean islands, mainly due to a lack of data. Here, we present a spatio-temporal analysis of the spread of chikungunya virus in Dominica, an island in the Western Hemisphere, using geo-referenced case data. The findings in this study suggest that females are at higher risk for chikungunya virus transmission in Dominica. In addition, there is statistically significant clustering of cases in densely populated areas. Lack of data prevented additional analyses on the impact of mosquito population density, environmental factors and housing conditions on the location and timing of the clusters. This study is relevant for chikungunya control in Dominica, and other regions can use similar methods to assess chikungunya risk at the local level.

## Introduction

Chikungunya is an acute febrile illness that can cause incapacitating joint pain, high fever and skin rash. There are no estimates of the global burden of chikungunya, however, country-specific estimates have been as high as 45.26 DALYs (Disability adjusted life years) per million for India, where it is endemic [1]. Chikungunya is caused by chikungunya virus (CHIKV), a mosquito-borne pathogen that is transmitted to humans primarily through the bite of infected *Aedes aegypti* and *Aedes albopictus* mosquitoes [2–4]. Symptoms typically appear after an incubation period of 3 to 7 days [2,5,6].

Over the last ten years, CHIKV has emerged and re-emerged in locations including Kenya (2004), Comoros (2004, 2007), Seychelles (2004, 2006), Mauritius (2005), La Reunion (2005, 2007), and India (2005) [4,5,7–9]. In July 2007, the first outbreak in a non-tropical region was reported in the Emilia-Romagna region in Italy [10] and in December 2013, the first autochthonous case of chikungunya in the Western Hemisphere was reported in St. Martin, an island in the Caribbean [11,12]. Due to human movement and abundance of *Aedes aegypti* mosquitoes in the Americas, an estimated one million people were infected with the virus within one year of its introduction [13].

The first case of CHIKV in Dominica, an island in the Caribbean, involved a 65-year-old woman from Good Hope, on the east coast of Dominica. The affected individual had travelled to St. Martin from December 9^th^ to 19^th^, 2013 and began experiencing symptoms on December 15^th^. Laboratory confirmation of diagnosis was received from the Caribbean Public Health Agency (CARPHA) on January 15^th^ 2014. Active surveillance of CHIKV cases began shortly thereafter (January 16^th^, 2014) [14,15]. Autochthonous transmission of CHIKV in Dominica was confirmed by CARPHA on January 25^th^, 2014 [14].

We present spatio-temporal analysis of the early spread of CHIKV in a country in the Western Hemisphere using geo-referenced chikungunya case data. We assess the following: (1) distribution of reported cases by sex and age; (2) the presence of statistically significant spatial and spatio-temporal clusters and (3) rate of virus transmission as indicated by distance and date of disease onset between clustered cases. Specifically, we focus on the first 417 cases reported in Dominica. Dynamical assessment of clustering during outbreaks would aid in the identification of high-risk locations for vector control.

## Materials and Methods

### Study Location

Dominica is a small island nation (750 sq. km) with an estimated population of 71,293 [16]. This volcanic island’s elevation ranges from sea level along the perimeter where the majority of the population resides to an altitude of 1,447 meters (Morne Santé) in the center of the island. The capital, Roseau, is located on the southwestern coast of the island and is the largest community with over 1/5 of the total island population [16]. There are two seasons: a wet season that runs from June to December and a dry season that runs from January to May. Dominica is administratively divided into ten parishes and is split into two health regions, which are further divided into seven health districts (Grand Bay, La Plaine, Roseau, Castle Bruce, St. Joseph, Marigot and Portsmouth) and fifty-two primary health centers.

### Case Definition

At the start of the CHIKV epidemic in Dominica, the following World Health Organization (WHO) case definition was used: *Suspected case*: *acute onset of fever >38*.*5°C and severe arthralgia/arthritis not explained by other medical conditions*, *and resides or has visited epidemic or endemic areas within 2 weeks prior to the onset of symptoms* [14,15]. However, once sustained local transmission had been established, the definition changed to: *Acute onset of fever (>38°C) and arthralgia/arthritis with or without headache*, *nausea*, *vomiting and atypical manifestation* [14]. Case confirmation was based on virus detection using real-time PCR, IgM ELISA and plaque-reduction neutralization test, as appropriate. Most infant cases had fever and skin rash and were either born into a family with CHIKV infection or a community with a CHIKV outbreak.

### Data Collection

The data provided by the Dominican Ministry of Health (MOH) was de-identified and each case was represented by a unique reference identification code. Information on all suspected cases was collected using a standardized questionnaire, which covered population demographics (age, sex), symptoms, geographic location (e.g., town or village) and occupation (reported for some cases). The geographic location and health district was reported for each case. With the aid of local public health workers and using a Global Positioning System (GPS) receiver, we successfully recorded the geographic coordinates of the home address for 417 of the first 500 cases of the outbreak. The data included cases with symptom onset from December 15^th^, 2013 to March 11^th^, 2014. No personal identifiers were present and maps presented in this paper do not identify patients’ houses.

### Analysis

We first summarized the distribution of cases across sex and age groups. To quantify the space-time interaction of individual reported chikungunya cases, we used the Knox method [17–19]. The method tests for interaction between cases with respect to distance and time, by comparing the observed to the expected number of cases in a specific space-time window. We selected distance and time intervals of 100m and 20 to 30 days range to account for the dispersal distance of *Ae*. *Aegypti* [17,20,21] and maximum sum of the CHIKV incubation period in both the vectors and humans, respectively. The critical chi-square values for the null hypothesis of spatial randomness were estimated based on 999 Monte Carlo simulations.

Based on the results from the Knox method, we applied a space-time permutation model and a Poisson purely spatial model to identify independent high-risk clusters and assess locations and timing of case clusters during the thirteen-week epidemic period. These methods have been used in clustering of cases to guide control programs during other infectious disease outbreaks [22–24]. The Poisson spatial model assumes that the number of cases in each town or village is Poisson distributed and under the null hypothesis, the risk and expected number of cases are proportional to the population size. Detailed description of the statistical methodology for the Poisson purely spatial model can be found in [25]. The space-time permutation algorithm performed with SaTScan 9.3.1 moves a circular scanning window over the study area, and evaluates thousands of overlapping scanning windows in space [23,26]. The height of each cylinder represents a time interval and the base is a geographical region around a centroid with a radius ranging from 1% to 50% of the population at risk [27]. The number of observed cases, the number of expected cases, and the Poisson generalized likelihood ratio (GLR) are estimated for each cylinder. The maximum GLR from the observed data is compared to the maximum GLRs from 999 random Monte Carlo simulations under the null hypothesis of no clustering. A p-value was used to indicate the statistical significance of each cluster and significance was assessed at the 0.05 level. The first analysis examined the clustering of cases occurring within a temporal window of 50% of the study period (default setting). The second analysis examined clustering within three day overlapping intervals (1–3 days, 2–4 days, 3–5 days, … 28–30 days) and five distances (100m, 200m, 300m, 400m, 500m) to respectively account for uncertainty in the reported date of illness onset, and assess robustness of selected distances [28]. We also included age and sex as covariates. Cases with identical coordinates were represented by a single location, resulting in 353 and 355 unique locations for the models with and without covariates. Statistical analysis and mappings were performed in SaTScan 9.3.1, R 2.10.1 (http://www.r-project.org/) and QGIS v2.4.

### Ethics Statement

The Institutional Review Board (IRB) at Boston Children’s Hospital approved this study.

## Results

### Case Description

**Fig 1:**
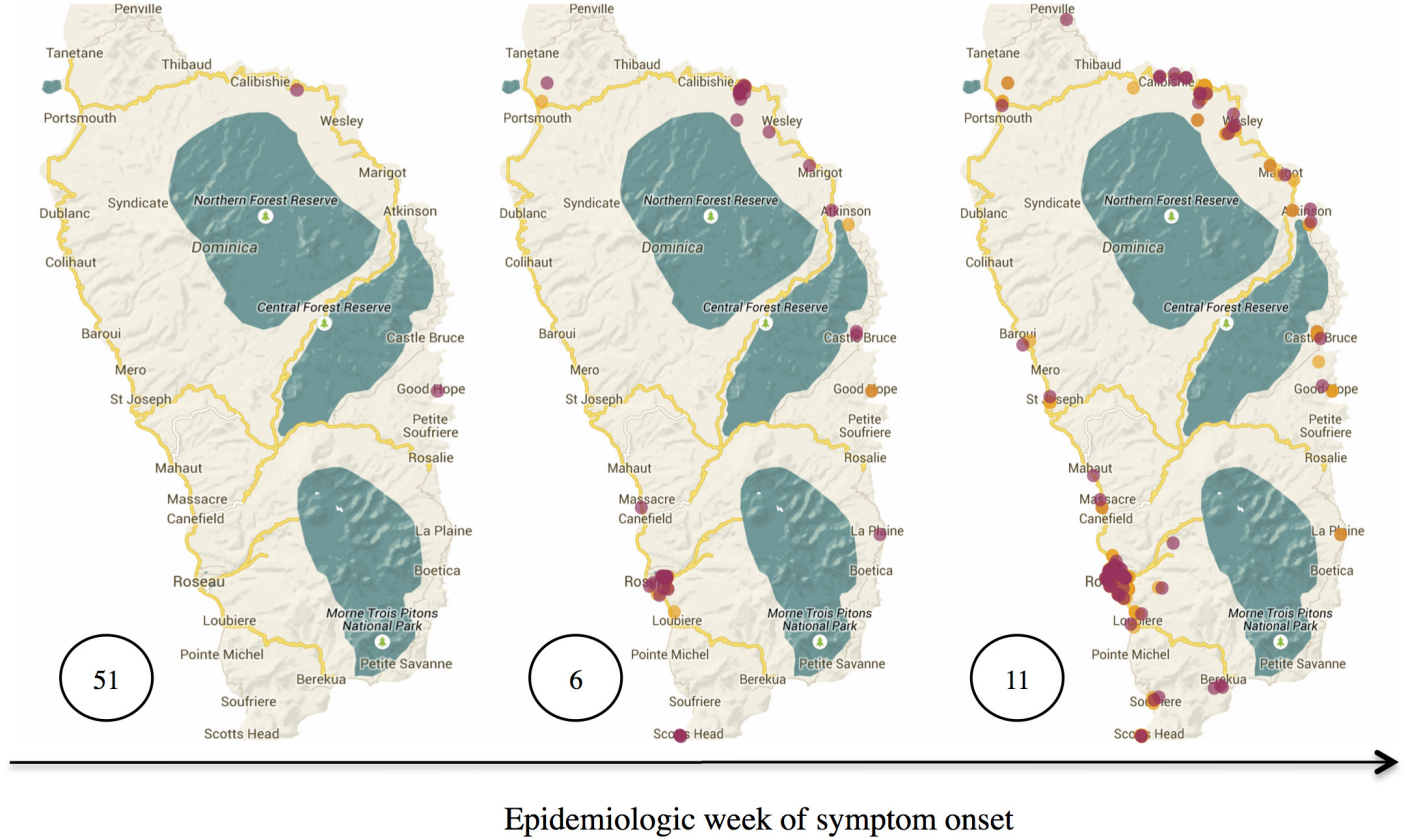
Spatial dispersion of chikungunya cases across the island of Dominica. The maroon dots represent new cases, while the orange dots represent previously reported cases. Data for 2013 and 2014 epidemiologic weeks 51, 6, and 11 are presented.

**Fig 2:**
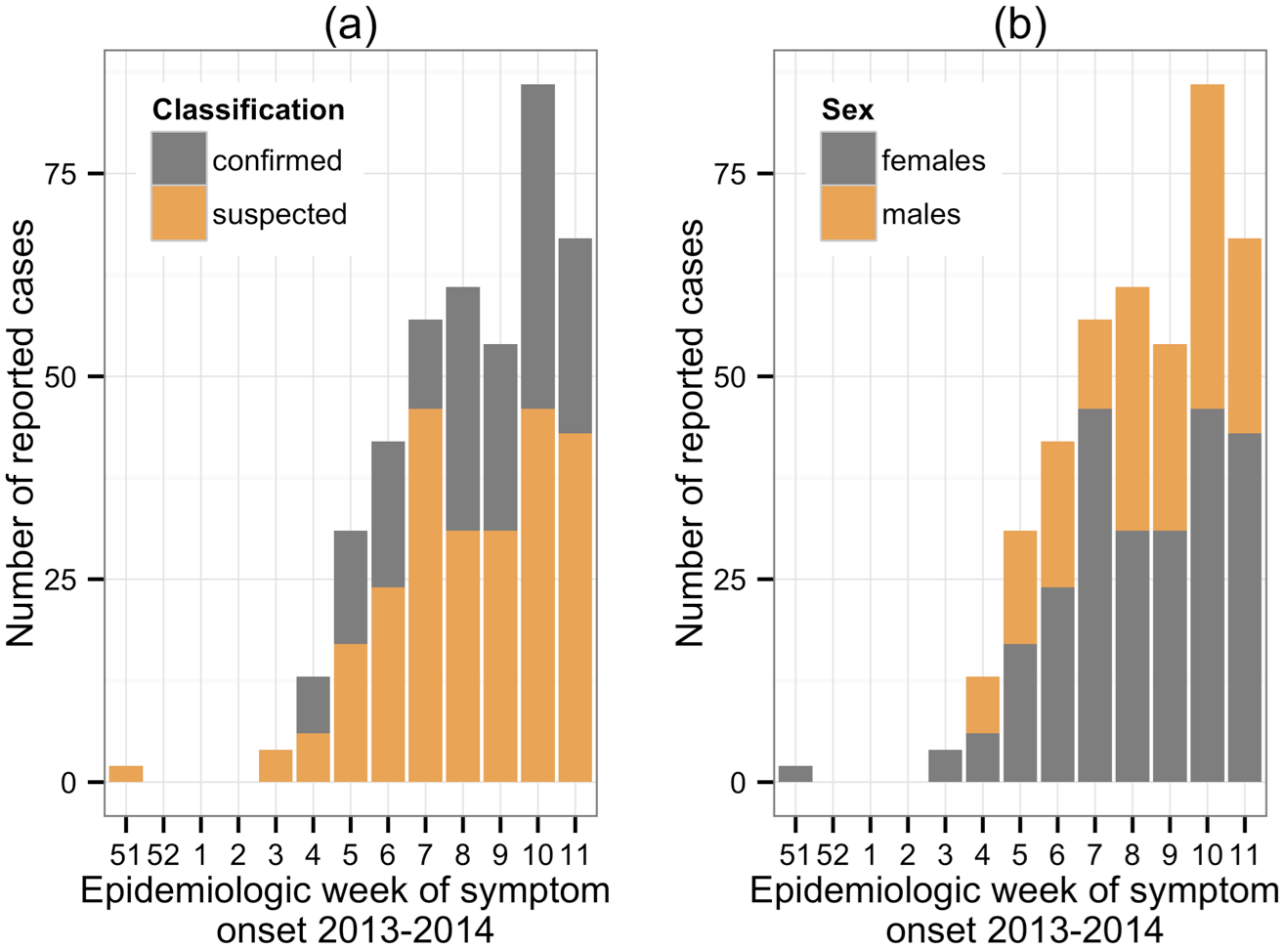
Epidemic curve by week of symptom onset; (a) case classification status and (b) sex. Reports start on epidemiologic week 51 of 2013 and end on week 11 of 2014.

Of the 417 cases (Figs 1 and 2), 66 were laboratory confirmed and 250 (60%) were female. The female to male odds ratio was 1.6, implying the odds of a reported case being female was 1.6 times the odds of being male. In addition, the male/female sex ratio was 0.67, which is similar to observations in other CHIKV studies [29–32]. The sex-specific incidence rates were 458.7 and 716.7 per 100,000 persons for males and females, respectively, despite a slightly higher number of males (36,411) than females (34,882) in the population [16]. This preponderance of female cases mostly concerned the age groups of 20–39 years (61.7% female vs. 38.3% male) and 40–59 (60.2% vs. 39.8%). The difference in the younger (19 years and less) and elderly populations (60 years and over) was less pronounced; 58.7% female vs. 41.3% male, and 57.1% vs. 42.9%, respectively. The median age for all cases was 33 years (min: 1, max: 92).

We also disaggregated the data into confirmed and suspected cases (Table 1). The disaggregated data had a male/female case ratio of approximately 0.57 and 0.69 for confirmed and suspected cases respectively. The distribution of cases across the various age groups was slightly different, however, a higher proportion of cases were in the under-40 age groups.

**Table 1:** Demographical characteristics of Chikungunya cases, Dominica, 2014.

Population Characteristics	Total	Confirmed	Suspected
	n (%)	n (%)	n (%)
Sex			
Male	167(40.0)	24(36.4)	143(40.7)
Female	250(60.0)	42(63.6)	208(59.3)
Age group			
19 years and less	104(1.2)	20(30.3)	84(23.9)
20–39 years	141(11.8)	18(27.3)	123(35.0)
40–59 years	118(28.3)	15(22.7)	103(29.3)
60 years and over	49(33.8)	13(19.7)	36(10.3)
Missing	5(24.9)	0(0)	5(1.4)

### Spatiotemporal Interaction between Cases

The Knox test indicated significant spatiotemporal interaction between cases (χ^2^ = 158.8; *P* < 0.001) with maximum distance and time fixed at 100m and 20 days respectively. As previously stated, we fixed the cluster detection to cases that were close in space (100m) and time (20 to 30 days) to reflect the biology of the system. The Knox test statistics was also statistically significant on all other days (21 to 30) with χ^2^ in the range 83.5 to 153.9, and *P* < 0.001.

### Spatial Clustering

**Fig 3:**
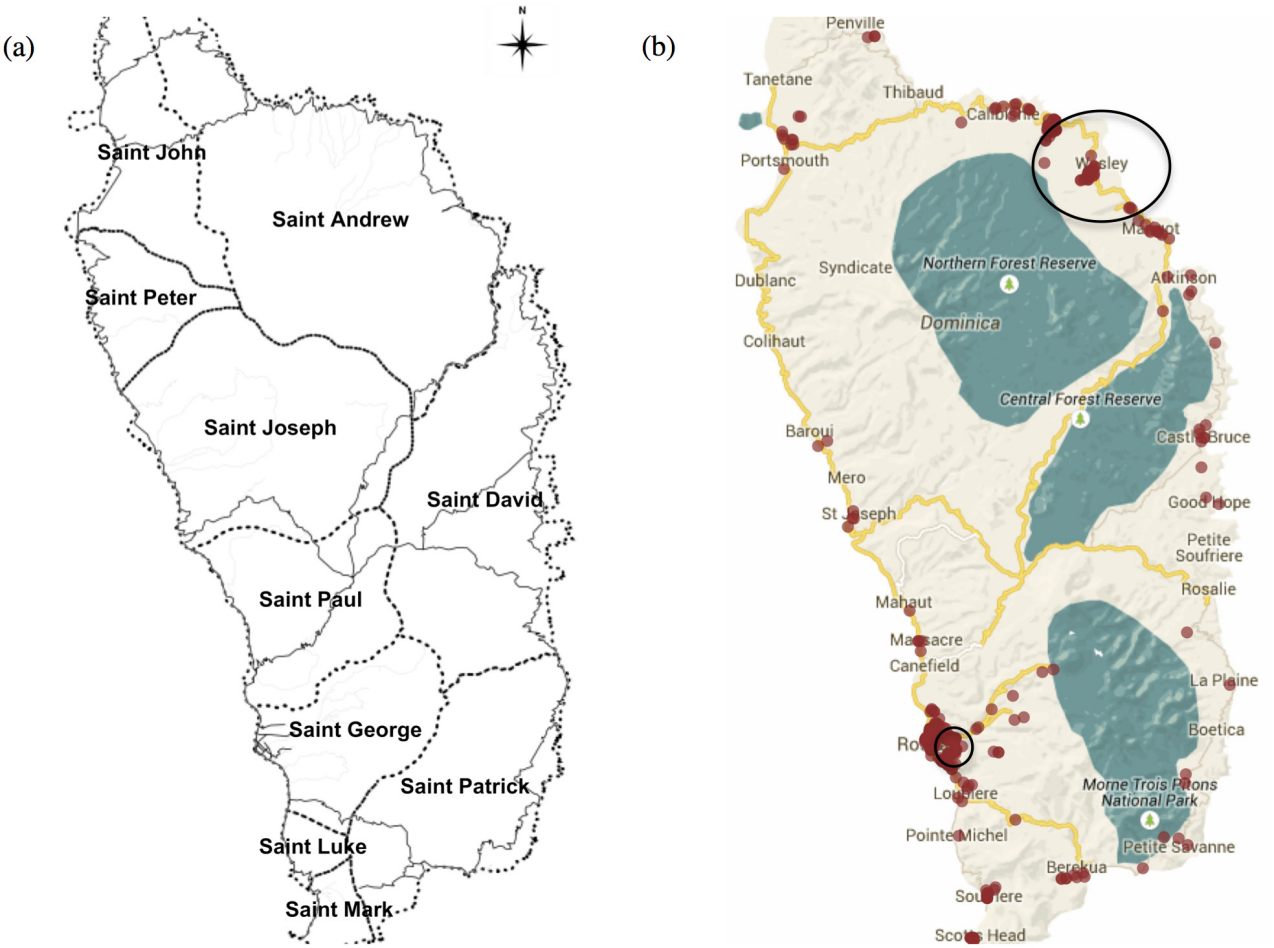
(a) Location of the ten Parishes of Dominica. (b) Spatial clusters identified by SaTScan. The clusters are located in St. Andrew and St. George Parishes.

SaTScan analysis detected two spatial clusters over the outbreak period using the Poisson model: one in the Bath Estate/Elmshall community (log likelihood ratio = 38.067, P < 0.001) and one in the Wesley, Woodford Hill community (log likelihood ratio = 35.222, P < 0.001) (Fig 3). The clusters contained 78 and 76 cases respectively, and the relative risk compared to the baseline was estimated at 3.51 and 3.37. Bath Estate/Elmshall and Wesley, Woodford Hill communities are located in St. George and St. Andrew Parishes, respectively.

### Spatio-temporal Clustering

**Fig 4:**
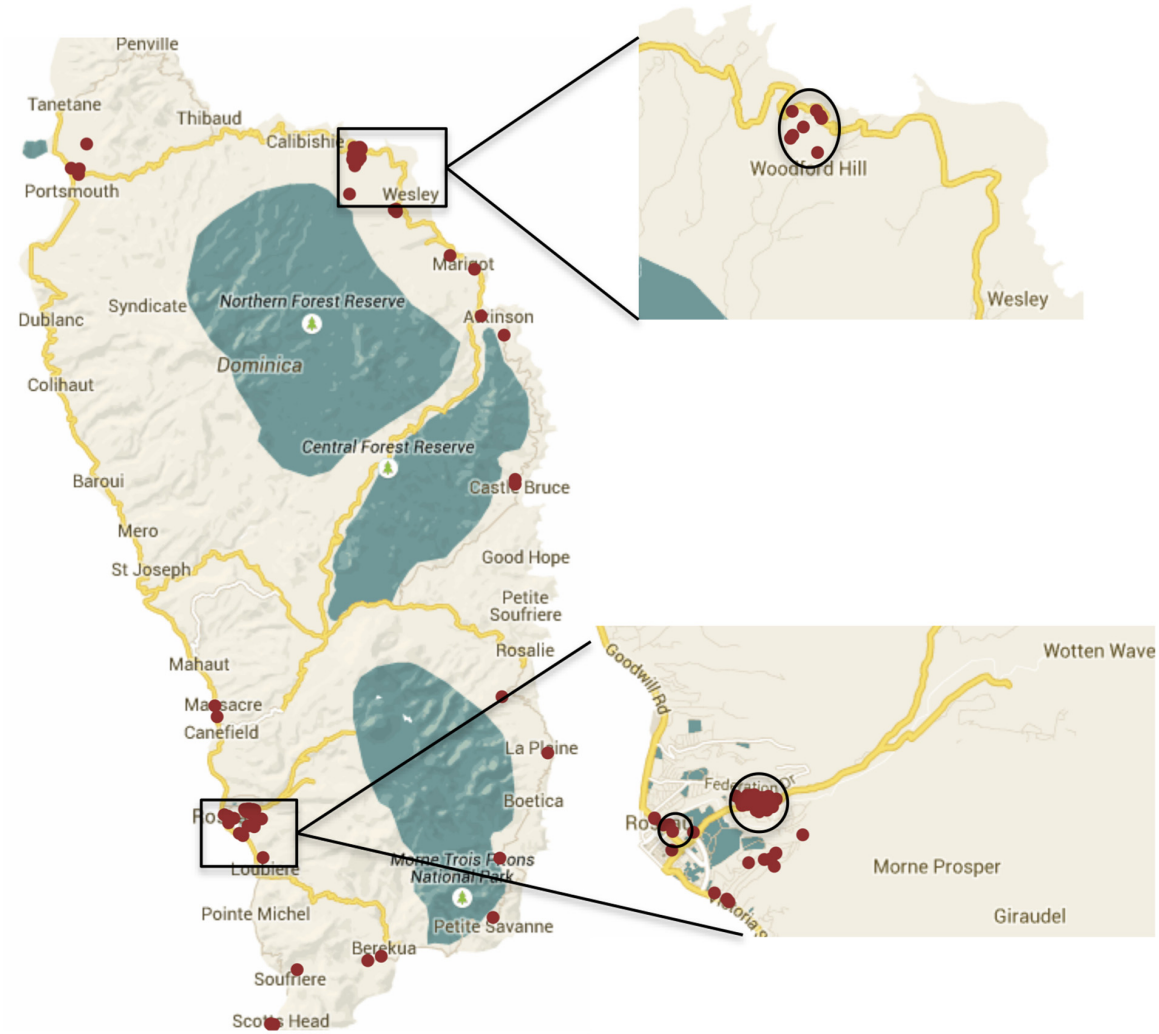
Three statistically significant space-time clusters identified by SaTScan. The clusters are located in St. George and St. Andrew Parishes.

The SaTScan space-time permutation model identified three statistically significant clusters (Fig 4). The primary cluster (P < 0.001) had 51 cases. The secondary clusters had 10 (P < 0.001) and 3 cases (P = 0.021). The clusters were located in the St. George, St. Andrew and St. George Parishes, respectively. The time frame for each cluster ranged from a single day to five weeks.

The space-time permutation model was adjusted for age and sex to account for the nonhomogeneous distribution across these groups (Table 1). Age had not been recorded for five patients so these cases were excluded from the analysis. With the addition of age and sex as covariates, two statistically significant clusters were identified. The primary cluster was the same as the cluster identified prior to age and sex adjustment. The secondary cluster (P < 0.001) in the St. George Parish was also similar to the previously identified cluster with two fewer cases.

### Clustering Rate

We assessed the rate of clustering by varying the temporal interval and distance between cases. For the models without covariates, statistically significant spatial clusters were detected at all temporal intervals (min: 2 days, max: 4 days). The temporal interval with the maximum number of spatial clusters was 1–3 days for cases within 100m. These clusters were independently observed on January 29–31, 2014 (26 cases, P < 0.001), February 10–12, 2014 (6 cases, P = 0.024), February 16, 2014 (3 cases, P = 0.036), and March 14, 2014 (3 cases, P = 0.005). In contrast, the maximum and minimum number of spatial clusters were 3 and 1, respectively, for models with covariates. Three clusters were observed for all spatial distances for temporal intervals 9–12 days; 5–10 days for 300m, 400m, and 500m and 11–14 days interval for cases within 100m. The clustering pattern was relatively consistent for the intervals 14–30 days for the models with and without covariates. The largest clusters for models with/without covariates were observed during the first few weeks of the outbreak.

## Discussion

This study presents spatio-temporal analysis of the early spread of CHIKV in a country in the Western Hemisphere using geo-referenced data. This constitutes an important step for understanding CHIKV spread in the Caribbean, and similar analysis in countries with ongoing CHIKV outbreaks will aid in assessing local CHIKV risk.

Our space-time analyses of the early spread of CHIKV in Dominica identified chikungunya case clusters and demonstrated heterogeneity of spread at the local level. Both the spatial and space-time analyses identified clusters in the St. George and St. Andrew Parishes. While the population density for St. George Parish is the highest at 964 persons per sq. mile, that of St. Andrew is much lower at 137 persons per sq. mile [16]. Population density has been considered a contributor to dengue epidemics [33,34], however, the cluster observations suggest that population density might impact the size but not the occurrence of clustering. Furthermore, two of the three space-time clusters preceded any vector control activities, suggesting some potential impact of vector control programs and education on the spread of CHIKV in Dominica. Additional analyses to investigate the impact of entomological and environmental factors on the cluster locations was not possible due to a lack of detailed entomological data, and unreliable land use and land cover data. Due to the length of the study period, we did not expect environmental covariates such as precipitation and temperature to have a major impact on the timing and location of clusters.

The strongest clustering in the temporal interval and spatial cluster analysis was observed for cases within 100m and with 1–3 days between reported symptom onsets. The spatial extent of 100m is consistent with the known dispersal distance of female *Ae*. *Aegypti* [17,20,21,28]. Additionally, there were 10 unique locations for the 23 cases in the largest cluster suggesting that multiple cases were in the same household. Reports of cases within close proximity over a short time period could be due to transmission by multiple mosquitoes that became infected at about the same time, or a single mosquito feeding on multiple nearby hosts [28,35–37].

The results also suggest that sex and age could also have some impact on the spread of CHIKV in Dominica. The incidence of female cases is much higher than male cases in total, as well as across different age groups. The higher number of female cases could be due to several factors including greater exposure and health seeking behavior [15], and socio-economic factors such as type and location of occupation. Unfortunately data on type and location of occupation was not available for most cases to further investigate these hypotheses.

There are a few factors that could have affected the findings of this study. First, individuals who experience mild illness, and asymptomatic cases (3%-25% [2,38–40]) are less likely to seek medical assistance and subsequently will not be captured by the surveillance system. To mitigate this limitation, active surveillance is needed and differentiation between travel-related and autochthonous transmission for the duration of the epidemic would be useful. In addition, clustering was solely based on patients’ household locations although transmission could have occurred at other locations such as schools and workplaces. Furthermore, the assumed circular shape of the clusters limits the identification of irregular clusters.

As of January 2014, the Environmental Health Department of Dominica has actively performed household inspections for all newly identified cases for vector control purposes, and treated bed nets from CARPHA were distributed starting in February 2014. Additionally, there has been fogging throughout the island and indoor residual spraying (IRS) at the homes of suspected cases [14,15]. Information on CHIKV prevention and vector control was also distributed through pamphlets, television and radio messages, social media, text messages, and community education sessions. These early interventions and surveillance efforts could have had an impact on the spread of CHIKV on the island.

Studies like this could be useful for early evaluation of case distribution and clustering to provide an assessment of risk at a finer geographical scale, identification of locations where vector control is most needed, and parameterization of CHIKV transmission models. Dynamic identification of clusters can lead to targeted local control efforts. However, it is difficult to conduct similar analyses for several of the CHIKV-affected nations in the Americas due to limitations in available data. Chikungunya surveillance can be improved by combining passive notification and active case detection. Consistent surveillance efforts, and detailed data are important to assess and control the spread of CHIKV. Additionally, resource-constrained regions can easily perform similar analysis due to the availability of open source software and geospatial data. Information presented in the paper can be used to guide future epidemiological studies to better understand the emergence of CHIKV in Dominica and elsewhere.

## References

[1] Krishnamoorthy K, Harichandrakumar KT, Krishna KA, Das LK. Burden of chikungunya in India: estimates of disability adjusted life years (DALY) lost in 2006 epidemic. J Vector Borne Dis.2009;46: 26–35. 19326705

[2] Staples JE, Breiman RF, Powers AM. Chikungunya fever: An epidemiological review of a re-emerging infectious disease. Clin Infect Dis.2009;49: 942–948. 10.1086/605496 19663604

[3] Thiberville S-D, Moyen N, Dupuis-Maguiraga L, Nougairede A, Gould EA, Roques P, et al Chikungunya fever: Epidemiology, clinical syndrome, pathogenesis and therapy. Antiviral Res. 2013; 99:345–370. 10.1016/j.antiviral.2013.06.009 23811281 PMC7114207

[4] Ng LFP, Ojcius DM. Chikungunya fever—Re-emergence of an old disease. Microbes Infect. 2009; 11: 1163–1164. 10.1016/j.micinf.2009.09.001 19737627

[5] Simon F, Savini H, Parola P. Chikungunya: A Paradigm of Emergence and Globalization of Vector-Borne Diseases. Med Clin North Am. 2008;92: 1323–1343. 10.1016/j.mcna.2008.07.008 19061754

[6] Tomasello D, Schlagenhauf P. Chikungunya and dengue autochthonous cases in Europe, 2007–2012. Travel Med Infect Dis. 2013;11: 274–284. 10.1016/j.tmaid.2013.07.006 23962447

[7] Pialoux G, Gaüzère B-A, Jauréguiberry S, Strobel M. Chikungunya, an epidemic arbovirosis. Lancet Infect Dis. 2007;7: 319–327. 17448935 10.1016/S1473-3099(07)70107-X

[8] Robinson M, Conan A, Duong V, Ly S, Ngan C, Buchy P, et al A Model for a Chikungunya Outbreak in a Rural Cambodian Setting: Implications for Disease Control in Uninfected Areas. PLoS Negl Trop Dis. 2014;8 10.1371/journal.pntd.0003120 PMC4161325 25210729

[9] Charrel R, de Lamballerie X, Raoult D. Chikungunya Outbreaks—The Globalization of Vectorborne Diseases. N Engl J Med. 2007;356: 769–71. 17314335 10.1056/NEJMp078013

[10] Rezza G, Nicoletti L, Angelini R, Romi R, Finarelli A, Panning M, et al Infection with chikungunya virus in Italy: an outbreak in a temperate region. Lancet. 2007;370: 1840–1846. 18061059 10.1016/S0140-6736(07)61779-6

[11] Sharp TM, Roth NM, Torres J, Ryff KR, Pérez Rodríguez NM, Mercado Chanis, et al Chikungunya Cases Identified Through Passive Surveillance and Household Investigations—Puerto Rico, May 5–August 12, 2014. MMWR Morb Mortal Wkly Rep. 2014;63: 1121–1128. 25474032 PMC4584601

[12] Morens DM, Fauci AS. Chikungunya at the Door—Déjà Vu All Over Again? N Engl J Med. 2014;371: 885–887. 10.1056/NEJMp1408509 25029435

[13] Johansson MA. Chikungunya on the move. Trends Parasitol. 2015;31: 43–5. 10.1016/j.pt.2014.12.008 25649340 PMC4583061

[14] Dominica Ministry of Health Weekly Chikungunya Report #1. 2014 Feb.

[15] Ahmed S, Francis L, Ricketts RP, Christian T, Polson-Edwards K, Olowokure B. Chikungunya Virus Outbreak, Dominica, 2014. Emerg Infect Dis. 2015;21. 10.3201/eid2105.141813 PMC4412235 25898214

[16] Commonwealth of Dominica: 2011 Population and Housing Census [Internet]. http://www.dominica.gov.dm/cms/files/2011_census_report.pdf

[17] Vazquez-Prokopec GM, Kitron U, Montgomery B, Horne P, Ritchie SA. Quantifying the Spatial Dimension of Dengue Virus Epidemic Spread within a Tropical Urban Environment. PLoS Negl Trop Dis. 2010;4:e920 10.1371/journal.pntd.0000920 21200419 PMC3006131

[18] Morrison AC, Getis A, Santiago M, Rigau-Perez JG, Reiter P. Exploratory space-time analysis of reported dengue cases during an outbreak in Florida, Puerto Rico, 1991–1992. Am J Trop Med Hyg. 1998;58: 287–298. 9546405 10.4269/ajtmh.1998.58.287

[19] Kulldorff M, Hjalmars U. The Knox Method and Other Tests for Space-Time Interaction. Biometrics. 1999;55: 544–552. 11318212 10.1111/j.0006-341x.1999.00544.x

[20] Harrington LC, Scott TW, Lerdthusnee K, Coleman RC, Costero A, Clark GG, et al Dispersal of the Dengue Vector Aedes Aegypti within and between Rural Communities. Am J Trop Med Hyg. 2005;72: 209–220. 15741559

[21] Russell RC, Webb CE, Williams CR, Ritchie SA. Mark–release–recapture study to measure dispersal of the mosquito Aedes aegypti in Cairns, Queensland, Australia. Med Vet Entomol. 2005;19: 451–457. 16336310 10.1111/j.1365-2915.2005.00589.x

[22] Elias Johannes, Harmsen Dag, Claus Heike, Hellenbrand Wiebke, Frosch Matthias, Vogel Ulrich. Spatiotemporal Analysis of Invasive Meningococcal Disease, Germany. Emerg Infect Dis. 2006;12(11):1689–95. 17283618 10.3201/eid1211.060682 PMC3372358

[23] Coulibaly D, Rebaudet S, Travassos M, Tolo Y, Laurens M, Kone A, et al Spatio-temporal analysis of malaria within a transmission season in Bandiagara, Mali. Malar J. 2013;12:82 10.1186/1475-2875-12-82 23452561 PMC3618208

[24] Coleman M, Coleman M, Mabuza A, Kok G, Coetzee M, Durrheim D. Using the SaTScan method to detect local malaria clusters for guiding malaria control programmes. Malar J. 2009;8:68 10.1186/1475-2875-8-68 19374738 PMC2679049

[25] Kulldorff M. A spatial scan statistic. Commun Stat Theor M. 1997;26: 1481–1496.

[26] Kulldorff M, Heffernan R, Hartman J, Assunção R, Mostashari F. A Space–Time Permutation Scan Statistic for Disease Outbreak Detection. PLoS Med. 2005; 2(3):e59 15719066 10.1371/journal.pmed.0020059 PMC548793

[27] Jones SG, Kulldorff M. Influence of Spatial Resolution on Space-Time Disease Cluster Detection. PLoS ONE. 2012;7(10):e48036 10.1371/journal.pone.0048036 23110167 PMC3480474

[28] Aldstadt J, Yoon I-K, Tannitisupawong D, Jarman RG, Thomas SJ, Gibbons RV, et al El análisis espacio-temporal de pacientes hospitalizados con dengue en áreas rurales de Tailandia revela importantes intervalos temporales en el patrón de transmisión del virus del dengue. Trop Med Int Health. 2012;17: 1076–1085. 10.1111/j.1365-3156.2012.03040.x 22808917 PMC4099473

[29] Caron M, Paupy C, Grard G, Becquart P, Mombo I, Nso BBB, et al Recent Introduction and Rapid Dissemination of Chikungunya Virus and Dengue Virus Serotype 2 Associated With Human and Mosquito Coinfections in Gabon, Central Africa. Clin Infect Dis. 2012;55: e45–e53. 10.1093/cid/cis530 22670036

[30] Vilain P, Larrieu S, Renault P, Baville M, Filleul L. How to explain the re-emergence of chikungunya infection in Reunion Island in 2010? Acta Trop. 2012;123: 85–90. 10.1016/j.actatropica.2012.03.009 22525433

[31] Laras K, Sukri NC, Larasati RP, Bangs MJ, Kosim R, Djauzi, et al Tracking the re-emergence of epidemic chikungunya virus in Indonesia. Trans R Soc Trop Med Hyg. 2005;99: 128–141. 15693148 10.1016/j.trstmh.2004.03.013

[32] Lam SK, Chua KB, Hooi PS, Rahimah MA, Kumari S, Tharmaratnam M, et al Chikungunya infection—an emerging disease in Malaysia. Southeast Asian J Trop Med Public Health. 2001;32: 447–51. 11944696

[33] Tauil PL. Urbanization and dengue ecology. Cadernos de Saude Publica. 2001;17 Suppl: 99–102. 11426270

[34] Chan T-C, Hu T-H, Hwang J-S. Daily forecast of dengue fever incidents for urban villages in a city. Int J Health Geogr. 2015;14(1): 9 10.1186/1476-072X-14-9 25636965 PMC4351941

[35] Scott TW, Chow E, Strickman D, Kittayapong P, Wirtz RA, Lorenz LH, et al Blood-Feeding Patterns of Aedes aegypti (Diptera: Culicidae) Collected in a Rural Thai Village. J Med Entomol. 1993;30: 922–927. 8254642 10.1093/jmedent/30.5.922

[36] Scott TW, Clark GG, Lorenz LH, Amerasinghe PH, Reiter P, Edman JD. Detection of Multiple Blood Feeding in Aedes aegypti (Diptera: Culicidae) During a Single Gonotrophic Cycle Using a Histologic Technique. J Med Entomol. 1993;30: 94–99. 8433350 10.1093/jmedent/30.1.94

[37] Gould DJ, Mount GA, Scanlon JE, Ford HR, Sullivan MF. Ecology and Control of Dengue Vectors on an Island in the Gulf of Thailand. J Med Entomol. 1970;7: 499–508. 5530313 10.1093/jmedent/7.4.499

[38] Queyriaux B, Simon F, Grandadam M, Michel R, Tolou H, Boutin J-P. Clinical burden of chikungunya virus infection. Lancet Infect Dis. 2008;8: 2–3. 18156079 10.1016/S1473-3099(07)70294-3

[39] Sissoko D, Moendandze A, Malvy D, Giry C, Ezzedine K, Solet JL, et al Seroprevalence and Risk Factors of Chikungunya Virus Infection in Mayotte, Indian Ocean, 2005–2006: A Population-Based Survey. PLoS ONE. 2008;3: e3066 10.1371/journal.pone.0003066 18725980 PMC2518850

[40] Retuya TJA, Ting D L, Dacula B D, Lanada J M, Roque V G, Hugo C T, et al Chikungunya fever outbreak in an agricultural village in Indang, Cavite, Philippines. Philipp J Microbiol Infect Dis. 1998;27: 93–96.

